# *catena*-Poly[[octa­aqua­bis­(μ_4_-benzene-1,3,5-tricarboxyl­ato)trizinc] tetra­hydrate]

**DOI:** 10.1107/S160053681101436X

**Published:** 2011-04-22

**Authors:** Lin Sun, Tao Run Qiu, Hong Deng

**Affiliations:** a School of Chemistry and Environment, South China Normal University, Guangzhou 510006, People’s Republic of China

## Abstract

In the title compound, {[Zn_3_(C_9_H_3_O_6_)_2_(H_2_O)_8_]·4H_2_O}_*n*_, there are two crystallographically independent Zn^II^ ions. One presents a trigonal-bipyramidal coordination geometry defined by five O atoms [three from two carboxyl­ate groups of two benzene-1,3,5-tricarboxyl­ate (BTC) ligands and the other two deriving from three water mol­ecules], while the other lies on an inversion centre and exists in a slightly distorted octa­hedral coordination geometry defined by six O atoms (two from two carboxyl­ate groups of two BTC ligands and the others from four water mol­ecules). A three-dimensional framework is further strengthened *via* O—H⋯O hydrogen-bonding inter­actons.

## Related literature

For background to the applications of metal–organic frameworks, see: Batten & Murray (2003 ▶); Zhong *et al.* (2008 ▶); Qiu *et al.* (2010 ▶). For the applications of benzene-1,3,5-tricarboxyl­ate, see: Yaghi *et al.* (1997 ▶); Xu *et al.* (2008 ▶); Xu *et al.* (2007 ▶); Liang *et al.* (2009 ▶); Wang *et al.* (2009 ▶). For compounds exhibiting similar Zn—O distances, see: Hua *et al.* (2010 ▶); Chen *et al.* (2010 ▶); Yang *et al.* (2008 ▶); Xu *et al.* (2007 ▶).
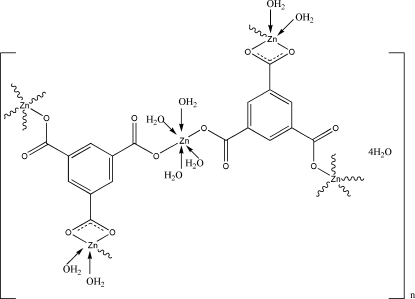


## Experimental

### 

#### Crystal data

[Zn_3_(C_9_H_3_O_6_)_2_(H_2_O)_8_]·4H_2_O
*M* *_r_* = 826.59
Monoclinic, 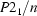

*a* = 14.745 (2) Å
*b* = 6.7960 (12) Å
*c* = 15.183 (3) Å
β = 94.543 (2)°
*V* = 1516.7 (4) Å^3^
*Z* = 2
Mo *K*α radiation
μ = 2.45 mm^−1^
*T* = 296 K
0.27 × 0.24 × 0.23 mm

#### Data collection

Bruker SMART APEX CCD diffractometer
Absorption correction: multi-scan (*SADABS*; Sheldrick, 1996 ▶) *T*_min_ = 0.521, *T*_max_ = 0.569
7485 measured reflections
2729 independent reflections
1990 reflections with *I* > 2σ(*I*)
*R*_int_ = 0.047

#### Refinement

*R*[*F* ^2^ > 2σ(*F* ^2^)] = 0.064
*wR*(*F* ^2^) = 0.225
*S* = 1.13
2729 reflections
205 parameters
1 restraint
H-atom parameters constrained
Δρ_max_ = 1.89 e Å^−3^
Δρ_min_ = −0.90 e Å^−3^

### 

Data collection: *APEX2* (Bruker, 2004 ▶); cell refinement: *SAINT* (Bruker, 2004 ▶); data reduction: *SAINT*; program(s) used to solve structure: *SHELXS97* (Sheldrick, 2008 ▶); program(s) used to refine structure: *SHELXL97* (Sheldrick, 2008 ▶); molecular graphics: *SHELXTL* (Sheldrick, 2008 ▶); software used to prepare material for publication: *SHELXTL*.

## Supplementary Material

Crystal structure: contains datablocks I, global. DOI: 10.1107/S160053681101436X/zk2004sup1.cif

Structure factors: contains datablocks I. DOI: 10.1107/S160053681101436X/zk2004Isup2.hkl

Additional supplementary materials:  crystallographic information; 3D view; checkCIF report

## Figures and Tables

**Table 1:** Hydrogen-bond geometry (Å, °)

*D*—H⋯*A*	*D*—H	H⋯*A*	*D*⋯*A*	*D*—H⋯*A*
O4*W*—H4*WB*⋯O6^i^	0.85	1.97	2.768 (9)	155
O5*W*—H5*WA*⋯O2^ii^	0.85	1.99	2.842 (9)	179
O6*W*—H6*WB*⋯O6^iii^	0.84	2.29	3.058 (13)	153
O5*W*—H5*WB*⋯O6^iv^	0.85	2.59	3.356 (12)	150

Symmetry codes: (i) 
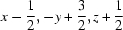
; (ii) 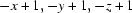; (iii) 
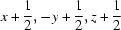
; (iv) 
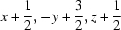
.

**Fig. 1.**
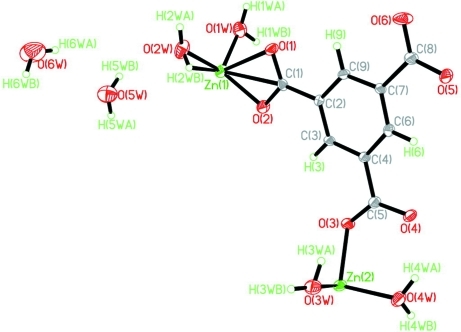
The structure of (I), showing the atomic numbering scheme. Non-H atoms are shown as 30% probability displacement ellipsoids.

**Fig. 2.**
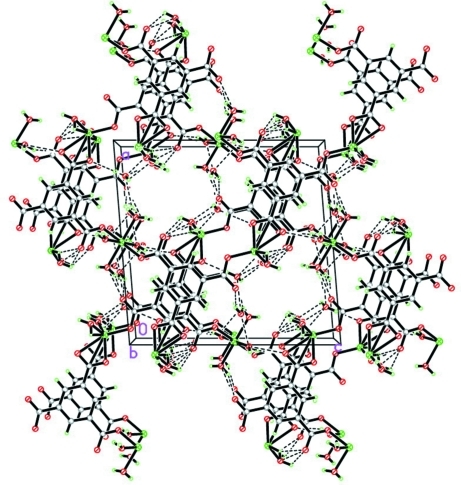
A packing view of (I) along the b axis, showing the O—H···O hydrogen bonds.

## supplementary crystallographic information

### Comment

The exploring of metal-organic frameworks (MOFs) has attracted considerable attention not only owing to their intriguing structral architectures and topologies, but also because of their many potential applications in catalysis, ion exchange, and magnetic, optical, and porous materials (Batten & Murray, 2003; Zhong *et al.*, 2008; Qiu *et al.*,2010). 1,3,5-benzenetricarboxylate with six O atoms from its three carboxylate groups is a good choice of O-donor ligand. And such ligand has been widely used to synthesize metal compounds (Yaghi *et al.*, 1997; Xu *et al.*, 2008; Xu *et al.*, 2007; Liang *et al.*, 2009; Wang *et al.*, 2009). Thus, we synthesize a new three-dimensional Zn-BTC metal-organic compound, {[Zn_3_(BTC)_2_(H_2_O)_8_](H_2_O)_4_}, with achiral channels along *b* direction, which was generated by the reaction of zinc sulfate heptahydrate, 1,3,5-benzenetricarboxylic acid and water at 150°C for 3 days.

There are two kinds of zinc atoms in the title compound (I) (Fig. 1). One is surrounded by five O atoms (three from two carboxylate groups of two BTC ligands and the other two deriving from three water molecules), exhibiting a trigonal bipyramidal geometry, the other is coordinated with six O atoms (two from two carboxylate groups of two BTC ligands; the others from four water molecules) and displays a slightly distorted octahedral geometry. All the BTC ligands have the same coordinated modes and each ligand coordinated to three zinc atoms. The bond distances of Zn—O_chelated carboxylate_ range from 1.999 (5)Å to 2.412 (6) Å. While the bond lengthes of Zn—O_monodentate carboxylate_ fall between 1.946 (6)Å and 2.049 (5) Å. And the Zn—O_w_ distances are in the normal range of 1.965 (6)–2.150 (6)Å (Table 1). All the distances of Zn—O in compound (I) are comparable to those found in the literatures (Hua *et al.*, 2010; Chen *et al.*, 2010; Yang *et al.*, 2008; Xu *et al.*, 2007). And there are weak interactions between Zn1 and C1 with the distances of 2.554Å and Zn1 and H2WB with with the distances of 2.0711 Å. A three-dimensional architecture is strengthened by the extended O—H···O hydrogen-bonding interactions (Table 2, Fig. 2)

### Experimental

A mixture of zinc sulfate heptahydrate (0.287 g; 1 mmol), benzenetricarboxylic acid (0.210 g; 1 mmol) and water (10 ml) was sealed in a 23 ml Teflon-lined stainless steel reactor and heated at 120°C under autogenous pressure for 72 h. Then the mixture was cooled down to room temperature at a rate of 5°C per hour, and colorless block crystals were obtained in a yield of 49% based on Zn

### Refinement

water H atoms were located in a difference Fourier map and were refined isotropically, Other H-atoms on aromatic ring were placed in calculated positions with C—H = 0.93 Å; refined using a riding model with *U*_iso_(H) = 1.2 *U*_eq_(C).

### Crystal data

**Table:**

[Zn_3_(C_9_H_3_O_6_)_2_(H_2_O)_8_]·4H_2_O	*F*(000) = 840.0
*M**_r_* = 826.59	*D*_x_ = 1.810 Mg m^−^^3^
Monoclinic, *P*2_1_/*n*	Mo *K*α radiation, λ = 0.71073 Å
Hall symbol: -P 2yn	Cell parameters from 2729 reflections
*a* = 14.745 (2) Å	θ = 2.0–25.2°
*b* = 6.7960 (12) Å	µ = 2.45 mm^−^^1^
*c* = 15.183 (3) Å	*T* = 296 K
β = 94.543 (2)°	Block, colourless
*V* = 1516.7 (4) Å^3^	0.27 × 0.24 × 0.23 mm
*Z* = 2	

### Data collection

**Table:**

Bruker SMART APEX CCD diffractometer	2729 independent reflections
Radiation source: fine-focus sealed tube	1990 reflections with *I* > 2σ(*I*)
graphite	*R*_int_ = 0.047
ω scans	θ_max_ = 25.2°, θ_min_ = 2.0°
Absorption correction: multi-scan (*SADABS*; Sheldrick, 1996)	*h* = −17→17
*T*_min_ = 0.521, *T*_max_ = 0.569	*k* = −8→8
7485 measured reflections	*l* = −18→11

### Refinement

**Table:**

Refinement on *F*^2^	Primary atom site location: structure-invariant direct methods
Least-squares matrix: full	Secondary atom site location: difference Fourier map
*R*[*F*^2^ > 2σ(*F*^2^)] = 0.064	Hydrogen site location: inferred from neighbouring sites
*wR*(*F*^2^) = 0.225	H-atom parameters constrained
*S* = 1.13	*w* = 1/[σ^2^(*F*_o_^2^) + (0.124*P*)^2^ + 4.534*P*] where *P* = (*F*_o_^2^ + 2*F*_c_^2^)/3
2729 reflections	(Δ/σ)_max_ = 0.002
205 parameters	Δρ_max_ = 1.89 e Å^−^^3^
1 restraint	Δρ_min_ = −0.90 e Å^−^^3^

### Special details

**Table:**

Geometry. All e.s.d.'s (except the e.s.d. in the dihedral angle between two l.s. planes) are estimated using the full covariance matrix. The cell e.s.d.'s are taken into account individually in the estimation of e.s.d.'s in distances, angles and torsion angles; correlations between e.s.d.'s in cell parameters are only used when they are defined by crystal symmetry. An approximate (isotropic) treatment of cell e.s.d.'s is used for estimating e.s.d.'s involving l.s. planes.
Refinement. Refinement of *F*^2^ against ALL reflections. The weighted *R*-factor *wR* and goodness of fit *S* are based on *F*^2^, conventional *R*-factors *R* are based on *F*, with *F* set to zero for negative *F*^2^. The threshold expression of *F*^2^ > σ(*F*^2^) is used only for calculating *R*-factors(gt) *etc*. and is not relevant to the choice of reflections for refinement. *R*-factors based on *F*^2^ are statistically about twice as large as those based on *F*, and *R*- factors based on ALL data will be even larger.

### Fractional atomic coordinates and isotropic or equivalent isotropic displacement parameters (Å^2^)

**Table:**

	*x*	*y*	*z*	*U*_iso_*/*U*_eq_	
C1	0.3865 (5)	0.7336 (11)	0.2885 (5)	0.0258 (16)	
C2	0.2896 (5)	0.7366 (11)	0.2512 (5)	0.0247 (16)	
C3	0.2199 (5)	0.7180 (11)	0.3061 (5)	0.0229 (15)	
H3	0.2334	0.7052	0.3667	0.027*	
C4	0.1296 (5)	0.7180 (11)	0.2720 (5)	0.0251 (16)	
C5	0.0531 (5)	0.6843 (12)	0.3308 (5)	0.0292 (17)	
C6	0.1136 (4)	0.7328 (10)	0.1806 (5)	0.0266 (17)	
H6	0.0539	0.7242	0.1561	0.032*	
C7	0.1800 (5)	0.7589 (11)	0.1259 (5)	0.0241 (16)	
C8	0.1573 (5)	0.7876 (13)	0.0286 (5)	0.0334 (19)	
C9	0.2690 (5)	0.7583 (11)	0.1620 (5)	0.0240 (15)	
H9	0.3160	0.7728	0.1251	0.029*	
O1	0.4494 (4)	0.7356 (9)	0.2381 (4)	0.0397 (15)	
O2	0.4054 (4)	0.7247 (9)	0.3705 (4)	0.0353 (13)	
O3	0.0741 (4)	0.6348 (9)	0.4086 (3)	0.0358 (13)	
O4	−0.0276 (4)	0.7041 (10)	0.2973 (4)	0.0398 (15)	
O5	0.0728 (4)	0.7813 (10)	0.0033 (4)	0.0407 (15)	
O6	0.2183 (4)	0.8087 (13)	−0.0218 (4)	0.064 (2)	
O1W	0.5996 (4)	0.9590 (10)	0.3383 (4)	0.0472 (16)	
H1WA	0.6200	1.0116	0.2931	0.071*	
H1WB	0.5642	1.0416	0.3600	0.071*	
O2W	0.6025 (4)	0.4905 (9)	0.3245 (4)	0.0504 (17)	
H2WA	0.6226	0.4688	0.2739	0.076*	
H2WB	0.5518	0.4334	0.3252	0.076*	
O3W	0.0151 (4)	0.2213 (10)	0.4358 (4)	0.0450 (15)	
H3WA	0.0380	0.2934	0.3975	0.067*	
H3WB	0.0384	0.1073	0.4327	0.067*	
O4W	−0.1226 (4)	0.5373 (11)	0.4216 (4)	0.0475 (17)	
H4WA	−0.1203	0.6053	0.3750	0.071*	
H4WB	−0.1606	0.5920	0.4533	0.071*	
O5W	0.6697 (5)	0.2093 (11)	0.4653 (5)	0.062 (2)	
H5WA	0.6465	0.2278	0.5141	0.092*	
H5WB	0.6894	0.3201	0.4485	0.092*	
O6W	0.8488 (7)	0.0366 (15)	0.4616 (8)	0.114 (4)	
H6WA	0.8232	0.1311	0.4498	0.172*	
H6WB	0.8175	−0.0498	0.4851	0.172*	
Zn1	0.54125 (6)	0.71412 (16)	0.37644 (6)	0.0342 (4)	
Zn2	0.0000	0.5000	0.5000	0.0330 (4)	

### Atomic displacement parameters (Å^2^)

**Table:**

	*U*^11^	*U*^22^	*U*^33^	*U*^12^	*U*^13^	*U*^23^
C1	0.024 (4)	0.027 (4)	0.025 (4)	−0.002 (3)	−0.004 (3)	0.004 (3)
C2	0.019 (4)	0.029 (4)	0.026 (4)	0.003 (3)	0.002 (3)	0.005 (3)
C3	0.024 (4)	0.030 (4)	0.015 (3)	0.000 (3)	0.003 (3)	0.002 (3)
C4	0.023 (4)	0.036 (4)	0.016 (4)	0.001 (3)	0.003 (3)	0.002 (3)
C5	0.023 (4)	0.044 (5)	0.020 (4)	0.001 (3)	0.002 (3)	0.000 (3)
C6	0.019 (4)	0.036 (4)	0.024 (4)	−0.004 (3)	−0.005 (3)	0.001 (3)
C7	0.021 (4)	0.034 (4)	0.016 (4)	−0.002 (3)	−0.001 (3)	0.005 (3)
C8	0.027 (4)	0.054 (5)	0.020 (4)	−0.002 (4)	0.001 (3)	0.002 (3)
C9	0.018 (3)	0.036 (4)	0.018 (4)	−0.003 (3)	0.003 (3)	0.000 (3)
O1	0.018 (3)	0.068 (4)	0.033 (3)	0.001 (3)	0.002 (2)	0.007 (3)
O2	0.024 (3)	0.057 (4)	0.024 (3)	−0.001 (3)	−0.005 (2)	0.000 (2)
O3	0.027 (3)	0.062 (4)	0.018 (3)	−0.003 (3)	0.002 (2)	0.009 (3)
O4	0.020 (3)	0.071 (4)	0.030 (3)	0.002 (3)	0.004 (2)	0.010 (3)
O5	0.020 (3)	0.077 (4)	0.024 (3)	0.000 (3)	−0.006 (2)	0.005 (3)
O6	0.030 (3)	0.139 (7)	0.022 (3)	−0.021 (4)	0.000 (3)	0.012 (4)
O1W	0.041 (3)	0.060 (4)	0.038 (4)	0.001 (3)	−0.010 (3)	0.001 (3)
O2W	0.039 (4)	0.059 (4)	0.051 (4)	0.009 (3)	−0.007 (3)	−0.017 (3)
O3W	0.045 (4)	0.056 (4)	0.035 (3)	0.003 (3)	0.013 (3)	0.001 (3)
O4W	0.029 (3)	0.080 (5)	0.034 (3)	0.006 (3)	0.004 (3)	0.006 (3)
O5W	0.073 (5)	0.064 (5)	0.048 (4)	−0.006 (4)	0.011 (4)	−0.012 (3)
O6W	0.105 (8)	0.080 (7)	0.166 (11)	−0.032 (6)	0.057 (8)	−0.003 (7)
Zn1	0.0231 (5)	0.0556 (7)	0.0228 (6)	0.0024 (4)	−0.0043 (4)	−0.0048 (4)
Zn2	0.0246 (7)	0.0539 (9)	0.0211 (7)	0.0012 (6)	0.0059 (5)	0.0050 (6)

### Geometric parameters (Å, °)

**Table:**

C1—O1	1.248 (9)	O1W—Zn1	1.981 (7)
C1—O2	1.256 (9)	O1W—H1WA	0.8505
C1—C2	1.495 (10)	O1W—H1WB	0.8502
C2—C9	1.372 (11)	O2W—Zn1	1.965 (6)
C2—C3	1.380 (10)	O2W—H2WA	0.8584
C3—C4	1.390 (10)	O2W—H2WB	0.8429
C3—H3	0.9300	O3W—Zn2	2.150 (6)
C4—C6	1.392 (10)	O3W—H3WA	0.8498
C4—C5	1.511 (10)	O3W—H3WB	0.8494
C5—O3	1.244 (9)	O4W—Zn2	2.099 (6)
C5—O4	1.263 (9)	O4W—H4WA	0.8483
C6—C7	1.344 (10)	O4W—H4WB	0.8518
C6—H6	0.9300	O5W—H5WA	0.8487
C7—C9	1.382 (10)	O5W—H5WB	0.8538
C7—C8	1.501 (10)	O6W—H6WA	0.7595
C8—O6	1.235 (10)	O6W—H6WB	0.8431
C8—O5	1.275 (10)	Zn1—O5^ii^	1.946 (6)
C9—H9	0.9300	Zn1—H2WB	2.0711
O1—Zn1	2.412 (6)	Zn2—O3^iii^	2.049 (5)
O2—Zn1	1.999 (5)	Zn2—O4W^iii^	2.099 (6)
O3—Zn2	2.049 (5)	Zn2—O3W^iii^	2.150 (6)
O5—Zn1^i^	1.946 (6)		
			
O1—C1—O2	119.4 (7)	Zn2—O3W—H3WB	152.2
O1—C1—C2	120.1 (7)	H3WA—O3W—H3WB	107.8
O2—C1—C2	120.5 (7)	Zn2—O4W—H4WA	116.8
C9—C2—C3	119.3 (7)	Zn2—O4W—H4WB	108.0
C9—C2—C1	120.4 (6)	H4WA—O4W—H4WB	107.7
C3—C2—C1	120.4 (7)	H5WA—O5W—H5WB	107.5
C2—C3—C4	120.8 (7)	H6WA—O6W—H6WB	114.2
C2—C3—H3	119.6	O5^ii^—Zn1—O2W	109.2 (3)
C4—C3—H3	119.6	O5^ii^—Zn1—O1W	101.6 (3)
C3—C4—C6	116.9 (6)	O2W—Zn1—O1W	108.0 (3)
C3—C4—C5	121.2 (6)	O5^ii^—Zn1—O2	101.8 (2)
C6—C4—C5	121.7 (6)	O2W—Zn1—O2	120.0 (2)
O3—C5—O4	124.5 (7)	O1W—Zn1—O2	114.4 (2)
O3—C5—C4	117.4 (7)	O5^ii^—Zn1—O1	159.2 (2)
O4—C5—C4	118.0 (7)	O2W—Zn1—O1	86.6 (2)
C7—C6—C4	123.5 (7)	O1W—Zn1—O1	85.5 (2)
C7—C6—H6	118.3	O2—Zn1—O1	57.8 (2)
C4—C6—H6	118.3	O5^ii^—Zn1—H2WB	111.6
C6—C7—C9	118.0 (7)	O2W—Zn1—H2WB	23.9
C6—C7—C8	120.6 (7)	O1W—Zn1—H2WB	128.1
C9—C7—C8	121.4 (7)	O2—Zn1—H2WB	96.9
O6—C8—O5	124.0 (7)	O1—Zn1—H2WB	77.4
O6—C8—C7	120.6 (7)	O3^iii^—Zn2—O3	180.000 (1)
O5—C8—C7	115.4 (7)	O3^iii^—Zn2—O4W	87.5 (2)
C2—C9—C7	121.4 (7)	O3—Zn2—O4W	92.5 (2)
C2—C9—H9	119.3	O3^iii^—Zn2—O4W^iii^	92.5 (2)
C7—C9—H9	119.3	O3—Zn2—O4W^iii^	87.5 (2)
C1—O1—Zn1	81.8 (4)	O4W—Zn2—O4W^iii^	180.000 (1)
C1—O2—Zn1	100.9 (5)	O3^iii^—Zn2—O3W^iii^	90.4 (2)
C5—O3—Zn2	131.1 (5)	O3—Zn2—O3W^iii^	89.6 (2)
C8—O5—Zn1^i^	116.7 (5)	O4W—Zn2—O3W^iii^	92.0 (3)
Zn1—O1W—H1WA	141.2	O4W^iii^—Zn2—O3W^iii^	88.0 (3)
Zn1—O1W—H1WB	98.5	O3^iii^—Zn2—O3W	89.6 (2)
H1WA—O1W—H1WB	107.6	O3—Zn2—O3W	90.4 (2)
Zn1—O2W—H2WA	134.0	O4W—Zn2—O3W	88.0 (3)
Zn1—O2W—H2WB	85.1	O4W^iii^—Zn2—O3W	92.0 (3)
H2WA—O2W—H2WB	107.6	O3W^iii^—Zn2—O3W	180.000 (1)
Zn2—O3W—H3WA	82.1		

Symmetry codes: (i) *x*−1/2, −*y*+3/2, *z*−1/2; (ii) *x*+1/2, −*y*+3/2, *z*+1/2; (iii) −*x*, −*y*+1, −*z*+1.

### Hydrogen-bond geometry (Å, °)

**Table:**

*D*—H···*A*	*D*—H	H···*A*	*D*···*A*	*D*—H···*A*
O4W—H4WB···O6^iv^	0.85	1.97	2.768 (9)	155.
O5W—H5WA···O2^v^	0.85	1.99	2.842 (9)	179.
O6W—H6WB···O6^vi^	0.84	2.29	3.058 (13)	153.
O5W—H5WB···O6^ii^	0.85	2.59	3.356 (12)	150.

Symmetry codes: (iv) *x*−1/2, −*y*+3/2, *z*+1/2; (v) −*x*+1, −*y*+1, −*z*+1; (vi) *x*+1/2, −*y*+1/2, *z*+1/2; (ii) *x*+1/2, −*y*+3/2, *z*+1/2.
